# Therapeutic applications of transcutaneous auricular vagus nerve stimulation with potential for application in neurodevelopmental or other pediatric disorders

**DOI:** 10.3389/fendo.2022.1000758

**Published:** 2022-10-12

**Authors:** Siyu Zhu, Xiaolu Zhang, Menghan Zhou, Keith M. Kendrick, Weihua Zhao

**Affiliations:** ^1^ The Clinical Hospital of Chengdu Brain Science Institute, Key Laboratory for NeuroInformation of Ministry of Education, Center for Information in Medicine, University of Electronic Science and Technology of China, Chengdu, China; ^2^ Institute of Electronic and Information Engineering of University of Electronic Science and Technology of China (UESTC) in Guangdong, Dongguan, China

**Keywords:** transcutaneous auricular vagus nerve stimulation, non-invasive, neural plasticity, pediatric disorders, protocol

## Abstract

Non-invasive transcutaneous auricular vagus nerve stimulation (taVNS) as a newly developed technique involves stimulating the cutaneous receptive field formed by the auricular branch of the vagus nerve in the outer ear, with resulting activation of vagal connections to central and peripheral nervous systems. Increasing evidence indicates that maladaptive neural plasticity may underlie the pathology of several pediatric neurodevelopmental and psychiatric disorders, such as autism spectrum disorder, attention deficit hyperactivity disorder, disruptive behavioral disorder and stress-related disorder. Vagal stimulation may therefore provide a useful intervention for treating maladaptive neural plasticity. In the current review we summarize the current literature primarily on therapeutic use in adults and discuss the prospects of applying taVNS as a therapeutic intervention in specific pediatric neurodevelopmental and other psychiatric disorders. Furthermore, we also briefly discuss factors that would help optimize taVNS protocols in future clinical applications. We conclude from these initial findings that taVNS may be a promising alternative treatment for pediatric disorders which do not respond to other interventions.

## 1 Introduction

Neural plasticity is a key mechanism involved in childhood brain development which both regulates and optimizes the function of neural circuitry controlling cognition and behavior. It can also help the brain to recover from injury (1–3). Maladaptive neuroplasticity may underlie the pathology of neurodevelopmental and other psychiatric disorders, such as autism spectrum disorder (ASD), anxiety, and depression (4, 5). Non-invasive brain stimulation (NIBS) techniques are increasingly used to promote neurological or psychiatric rehabilitation by modulating neural plasticity (6). In the last two decades, transcutaneous auricular vagus nerve stimulation (taVNS) has in particular attracted attention in clinical applications since Ventureyra (7) first proposed it as a non-invasive alternative to vagal nerve stimulation (VNS) for treatment of epilepsy (7). To date, taVNS has been used to help alleviate symptoms not only of epilepsy but also splanchnic diseases (e.g., heart failure) (8), stroke (9, 10) and tinnitus (11, 12) as well as some psychiatric disorders (e.g., major depressive disorder (MDD) (13–15). Increasing evidence from animal studies and clinical trials primarily in adult humans suggest that the therapeutic effects of invasive and noninvasive VNS may stem from its role in modulating maladaptive brain plasticity (10, 15–18). This may particularly be particular relevance in the case in developing child and adolescent brains given evidence from brain imaging that they are more highly plastic relative to adults (2, 19–21). Indeed, children and adolescents show accelerated neural plasticity compared to adults after brain stimulation (22).

There is a high prevalence of ASD (around 1%), attention-deficit/hyperactivity disorder (ADHD, 4%), disruptive behavioral disorder (DBD, 6.1%), obsessive-compulsive disorder (OCD, between 2% ~ 4%), depression and anxiety-related disorders (around 5%) in pediatric populations worldwide (23–26). Furthermore, overlapping clinical behavioral manifestations across these disorders and comorbid conditions are often reported (27–29). For example, social dysfunction is often seen in ASD, ADHD and obsessive-compulsive disorder (OCD) (30–32). Impulsivity and inattention are not only reported in ADHD, but also ASD and DBD (33, 34). The high frequency of comorbidities could be a result of shared pathophysiology and associated mechanisms. Importantly,, taVNS has been shown to have modulatory effects on cortical and subcortical brain regions that are associated with the neuropathology of these disorders and to help regulate some social-emotional functions that are impaired in them (35–39). These findings support the use of taVNS as a promising non-pharmaceutical treatment to mitigate symptoms of these disorders.

Currently, behavioral training is the most commonly used intervention technique for the aforementioned intractable neurodevelopmental and other psychiatric disorders that are prevalent during childhood/adolescence (40). For instance, language and social skill training are commonly used for children with ASD (41, 42). Additionally, cognitive behavioral therapy is frequently adopted as a treatment for depression (43). Intensive behavioral therapies may successfully improve behavioral outcomes in patients with these disorders by promoting adaptive plasticity in dysregulated neural circuitry (44, 45). However, these behavioral interventions are lengthy and time consuming, and a proportion of children fail to benefit. On the other hand, taVNS as a non-invasive technique has been recently reported to improve clinical outcomes in some intractable disorders, such as major depression disorder and post-traumatic stress disorder (46–49). In sum, these may suggest taVNS as a potential adjunctive non-invasive technique to help increase the benefit of behavioral interventions.

Here in the current review, we have therefore summarized current preliminary evidence for the effects of taVNS on different clinical behavioral manifestations targeting pediatric neurodevelopmental and other psychiatric disorders, including ASD, ADHD, OCD, DBD, depression and anxiety-related disorders and also briefly illustrate the underlying mechanisms of taVNS effects from the perspective of anatomical and neuroendocrine aspects of vagus nerve stimulation. In addition, we briefly discuss feasibility issues and several factors and that would help optimize taVNS protocols to improve therapeutic effects when applied in clinical situations in the future.

## 2 Anatomical and neuroendocrine mechanisms of action

**Figure 1:**
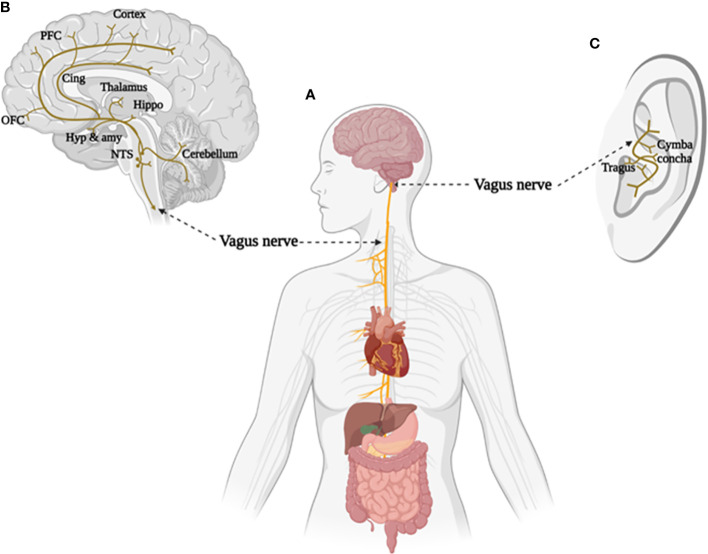
The brain and body projections of vagus nerve. **(A)** Illustration of the connection between brain and major body organs via the vagus nerve. **(B)** Areas of the brain involved in the afferent vagal pathway. Nucleus tractus solitarius (NTS), hypothalamus (Hyp), amygdala (amy), hippocampus (Hippo), cingulate cortex (Cing), orbital frontal cortex (OFC), and prefrontal cortex (PFC). **(C)** Distribution of the vagus nerve in the external ear. Created with BioRender.com.

The vagus nerve is the tenth cranial nerve that starts at the level of the brainstem and establishes a mutual connection between the brain and major body organs (**Figure 1A**). Afferent fibers of the vagus nerve send sensory (visceral and somatic) impulses to the vagal nuclei connections, the nucleus of the solitary tract (NST) and spinal nucleus of the trigeminal nerve (SNT), located in the medulla. Components of sensory information are further relayed to higher order brain regions (e.g. hippocampus, amygdala, thalamus and neocortex), thereby allowing the vagus nerve to modulate activity in widespread subcortical and cortical brain areas (50, 51). Thus, signals generated in the vagus nerve have the potential to affect a broad range of brain functions (see **Figure 1B**, for more detailed information regarding the physiology of the vagus nerve see (52)).

Interest in artificial VNS for therapeutic purpose has increased given the crucial role that the vagus plays in determining brain-body interactions. Evidence from animal models and clinical studies has demonstrated a potential for invasive VNS in modulating neural and physiological changes contributing to a number of chronic diseases (53, 54). Therefore, a large variety of disorders, such as epilepsy, migraine, inflammation maladaptive and metabolic syndrome are possible potential targets for VNS therapy (55). Anatomical evidence from humans and other animal species indicates that the tragus, concha, and cymba concha in the external auditory canal are the only places in the body with a cutaneous afferent vagus nerve distribution, making non-invasive transcutaneous stimulation of the vagus nerve possible (51, 56) (**Figure 1C**).

A number of brain imaging studies have shown that taVNS modulates brain function primarily by its direct afferent projections to specific brain structures, including the brainstem and other higher order relays of vagal afferents (visceral and somatic), such as the amygdala, hypothalamus and prefrontal cortex (50, 51, 57). Although the pathways by which taVNS exerts its various effects are still poorly understood, its potential for regulation of neurotransmission and promoting neuroplasticity is important in the context of neurodevelopmental and other psychiatric disorders. For instance, treatment effects of taVNS on stroke and tinnitus via its modulatory role in motor and sensory neural plasticity have been increasingly reported (9–12). Moreover, taVNS is also associated with the release of noradrenaline in the brain, as well as the inhibitory transmitter GABA, which potentially leads to VNS-mediated seizure reduction and antidepressant effects (58). Additionally, VNS inhibits excitatory glutamate release (59) and also increases the release of neurotrophic factors as well as stimulating cellular proliferation and neurogenesis in the brain, which correlate not only with antidepressant effects but also neuronal plasticity, memory, learning and cognitive processes (60).

## 3 Potential taVNS effects on clinical symptoms

Currently, taVNS has already been approved in Europe as a treatment for epilepsy and depression in 2020, for chronic pain in 2012 and for anxiety in 2019,and was also approved by the US Food and Drug Administration (FDA) for therapeutic use in depression and anxiety in 2006 (61, 62). Further, studies in healthy populations have demonstrated that taVNS can enhance cognitive performance (58) and brain-body functions (52), suggesting its potential therapeutic role in a number of disorders. We have therefore summarized the reported effects of taVNS on specific clinical symptoms in the following sections (also see in **Table 1**).

**Table 1:** Characteristics of task-related taVNS studies that are included in the review.

Study(author/year)	Sample size	Study design & protocol	Age(years)	Stimulationparameter	Symptom	Targetdisorder	taVNS effects
Colzato et al., 2018 (63)	taVNS: 40 Sham: 40 F: 50	Between Acute taVNS	17-33	taVNS: cymba conchae sham: earlobe 0.5mA, 25Hz, 200-300μs, 30s on 30s off	Depression	Depressive disorders	Divergent thinking **↑**
Neuser et al., 2020 (64)	81 (47 F)	Within Acute taVNS	25.3(3.8)	taVNS: cymba conchae 1.28(0.58) mA sham: earlobe 1.82(0.63) mA 25Hz, 30s on 30s off	Depression	Depressive disorders	Reward seeking **↑**
De Smet et al., 2021 (65)	taVNS: 42 Sham: 41 F: 66	Between Acute taVNS	21.11(3.10)	taVNS: cymba conchae 1.37(0.81) mA sham: earlobe 1.89(0.89) mA 25Hz, 250μs, 30s on 30s off	Depression	Depressive disorders	Negative emotion regulation **↑**
Ferstl et al., 2021 (66)	82 (47 F)	Within Acute taVNS	24.6(3.5)	taVNS: cymba conchae sham: earlobe 25Hz, 30s on 30s off	Depression	Depressive disorders	Mood recovery**↑**
Steenbergen et al., 2021 (67)	73 (58 F)	Within Acute taVNS	18-28	taVNS: cymba conchae sham: earlobe 0.5mA, 25Hz, 200-300μs, 30s on 30s off	Depression	Depressive disorders	Recognition of sadness **↓**
Koenig et al., 2021 (68)	33 (27 F) adolescents with major depressive disorders	Within Acute taVNS	14-17	taVNS: concha sham: earlobe 0.5mA, 1Hz, 250μs, 30s on 30s off	Depression	Depressive disorders	Attention to sad stimuli **↓**
Kraus et al., 2007 (38)	6 (5 F)	Within Acute taVNS	20-37	taVNS: inner tragus sham: earlobe 8Hz, 20μs	Depression	Depressive disorders	BOLD-signal in limbic brain areas**↓** Subjective well-being **↑**
Burger et al.,2020 (69) (Study 1)	taVNS: 45 Sham: 49 subclinical, high trait worrying sample	Between Acute taVNS	Not reported	taVNS: cymba conchae sham: earlobe 0.5mA, 25Hz, 250μs, 30s on 30s off	Anxiety and fear	GAD	Attentional engagement to threat **↓**
Burger et al., 2019 (70)	taVNS: 48 Sham: 49 subclinical, high trait worrying sample	Between Acute taVNS	Not reported	taVNS: cymba conchae sham: earlobe 0.5mA, 25Hz, 250μs, 30s ON 30s off	Anxiety and fear	GAD PTSD	Negative thought intrusions **↓**
Burger et al., 2017 (71)	taVNS: 25 Sham: 26 F: 26	Between Acute taVNS	20-36	taVNS: cymba conchae sham: earlobe 0.5mA, 25Hz, 250μs, 30s on 30s off	Anxiety and fear	Anxiety PTSD	Extinction of declarative fear **↑**
Burger et al., 2016 (72)	taVNS: 18 Sham: 13 F: 24	Between Acute taVNS	18-25	taVNS: cymba conchae sham: earlobe 0.5mA, 25Hz, 250μs, 30s on 30s off	Anxiety and fear	Anxiety PTSD	Extinction learning **↑**
Szeska et al., 2020 (73)	80 (57 F) Fear learning group: taVNS: 20 sham: 20 control group: taVNS: 20 sham: 20	Mixed Acute taVNS	18-34	taVNS: cymba conchae 2.28(1.13) mA sham: earlobe 2.53(1.11) mA, 25Hz, 200-300μs, 30s on 30s off	Anxiety and fear	Anxiety PTSD	Inhibition of fear potentiated startle responses **↑**
Jacobs et al., 2015 (74)	30 (15 F)	Within Acute taVNS	60.57(2.54)	taVNS: inner tragus sham: earlobe 5.0mA, 8Hz, 200μs	Anxiety and fear	Anxiety PTSD	Associated memory performance **↑**
Giraudier et al., 2020 (75)	60 (46 F)	Between Acute taVNS	23.45(4.87)	taVNS: cymba conchae 1.48(0.59) mA sham: earlobe 1.31(0.50) mA 25Hz, 200-300μs, 30s on 30s off	Anxiety and fear	Anxiety PTSD	Recollection-based memory**↑**
Colzato et al., 2017 (76)	38 (30 F)	Within Acute taVNS	18-26	taVNS: cymba conchae sham: earlobe 0.5mA,25Hz, 200-300μs, 30s on 30s off	Social dysfunction	ASD ADHD OCD	Recognition of emotions **↑**
Sellaro et al., 2018 (77)	24 (15 F)	Within Acute taVNS	18-28	taVNS: cymba conchae sham: earlobe 0.5mA, 25Hz, 200-300μs, 30s on 30s off	Social dysfunction	ASD ADHD OCD	Emotion recognition **↑**
Zhu et al., 2022 (78)	49 (17 F)	Within Acute taVNS	19.88(1.62)	taVNS: tragus 0.86(0.04) mA sham: earlobe 1.49(0.08) mA 25Hz, 500μs, 30s on 30s off	Social dysfunction	ASD ADHD OCD	Visual attention towards social salient facial features **↑** Endogenous oxytocin release **↑**
Koenig et al., 2021 (68)	30 (24 F) healthy controls	Within Acute taVNS	14-17	taVNS: concha sham: earlobe 0.5mA, 1Hz, 250μs, 30s on 30s off	Social dysfunction	ASD ADHD OCD	Emotion recognition **↑**
Villani et al., 2019 (79)	46 (32 F)	Within Acute taVNS	21.2(3.1)	taVNS: tragus 1.26(0.23) mA sham: earlobe 1.18(0.18) mA 25Hz, 250μs	Social dysfunction	Social dysfunction	Interoceptive accuracy **↑**
Maraver et al., 2020 (80)	43 (39 F)	Within Acute taVNS	18-30	taVNS: cymba conchae sham: earlobe 0.5mA, 25Hz, 200-300μs, 30s on 30s off	Social dysfunction	ASD ADHD OCD	Attention to faces with a direct gaze **↑**
Steenbergen et al., 2021 (67)	73 (58 F)	Within Acute taVNS	18-28	taVNS: cymba conchae sham: earlobe 0.5mA, 25Hz, 200-300μs, 30s on 30s off	Social dysfunction	ASD ADHD OCD	Recognition of anger **↑**
Ventura-Bort et al., 2021 (81)	37 (20 F)	Within Acute taVNS	23.15	taVNS: cymba conchae 1.34 mA sham: earlobe 1.58 mA 25Hz, 200-300μs	Social dysfunction	ASD ADHD OCD	Recollection-based memory for emotional material **↑** Attentional discrimination between emotional and neutral scenes**↑**
Steenbergen et al., 2015 (82)	30 (26 F) taVNS: 15 Sham: 15	Between Acute taVNS	18-27	taVNS: outer auditory canal sham: earlobe 0.5mA, 25Hz, 200-300μs, 30s on 30s off	Impulsivity and inattention	ADHD ASD DBD	Responses when two actions were executed in succession **↑**
Beste et al., 2016 (83)	51 (37 F) taVNS: 25 Sham: 26	Between Acute taVNS	23.63	taVNS: inner ear sham: earlobe 0.5mA, 25Hz 200-300μs, 30s on 30s off	Impulsivity and inattention	ADHD ASD DBD	The ability of inhibitory control **↑**
Fischer et al., 2018 (84)	21 (18 F)	Within Acute taVNS	20.3(1.4)	taVNS: cymba conchae 1.3 mA sham: earlobe 1.49 mA 25Hz, 200-300μs, 30s on 30s off	Impulsivity and inattention	ADHD ASD DBD	Adaption to conflict **↑**
Jongkees et al., 2018 (85)	40 (32 F) taVNS: 20 Sham: 20	Between Acute taVNS	taVNS: 22.3(2.7) years Sham: 22.5(2.5) years	taVNS: medial of the tragus sham: earlobe 0.5mA, 25Hz, 200-300μs, 30s on 30s off	Impulsivity and inattention	ADHD ASD DBD	Response selection processes **↑**
Keute et al., 2019 (86)	16 (8 F)	Within Acute taVNS	20-28	taVNS: cymba conchae 5.9(1.6) mA sham: earlobe 7.5(0.8) mA 25Hz, 200μs, 30s on 30s off	Impulsivity and inattention	ADHD ASD DBD	Automatic motor response inhibition **↑**
Keute et al., 2020 (87)	22 (16 F)	Within Acute taVNS	21-28	taVNS: cymba conchae 2.37(0.16) mA sham: earlobe 2.6 mA 25Hz, 200μs, 30s on 30s off	Impulsivity and inattention	ADHD ASD DBD	General adaptive control and sustained attention **↑**
Borges et al., 2020 (88)	23 (9 F)	Within Acute taVNS	23.17(4.08)	taVNS: cymba conchae 2.19(0.93) mA sham: earlobe 2.20(1.06) mA 25Hz, 200-300μs, 30s on 30s off	Impulsivity and inattention	ADHD ASD DBD	Cognitive flexibility **↑**
Pihlaja et al., 2020 (89)	25 (16 F)	Within Acute taVNS	25.5(4.8)	aVNS: inner tragus sham: earlobe 30Hz, 250μs	Impulsivity and inattention	ADHD ASD DBD	Cognitive control resources required to withhold a prepotent response **↓**
Steenbergen et al., 2020 (90)	84 (52 F)	Within Acute taVNS	22.32(2.71)	taVNS: cymba conchae sham: earlobe 0.5mA, 25Hz, 200-300μs, 30s on 30s off	Impulsivity and inattention	ADHD ASD DBD	Self-control **↑**
Llanos et al. 2020 (91)	36 (20 F) tVNS-hard: 12 tVNS-easy: 12 control: 12	Between Acute taVNS	21.60(3.56)	taVNS: cymba conchae hard: 1.67(0.79) mA easy: 1.24(0.88) mA control: no stimulation	Others/ Language deficits	ASD	Speech category learning & retention of correct stimulus response associations **↑**
Thakkar et al., 2020 (92)	37 (27 F) Computer control: 7 Device sham control: 7 Earlobe stimulation control: 9 taVNS: 14	Between Acute taVNS	18-28	taVNS: cymba conchae 1.68(0.87) mA sham control: cymba conchae no stimulation earlobe control: earlobe 1.51(0.35) mA 5Hz, 200μs, 30s on 30s off	Others/ Language deficits	ASD	Novel orthography acquisition **↑**
Hong et al., 2019 (93)	14 patients requiring open laparotomy (8 F)	Within Acute taVNS	57.6 (10.5)	taVNS: cymba conchar 10mA, 25Hz, 250μs, Control: no stimulation	Others/ Gastrointestinal problems	ASD	Stomach function **↑**
Teckentrup et al., 2020 (94)	22 (14 F)	Within Acute taVNS	19-29	taVNS: cymba conchae 1.37(0.81) mA sham: earlobe 1.89(0.89) mA 25Hz 30s on 30s off	Others/ Gastrointestinal problems	ASD	Gastric function **↑**
Steidel et al., 2021 (95)	HF taVNS: 24 (15 F) LF taVNS: 28 (18 F)	Mixed Acute taVNS	25.5(5.2)	taVNS: cymba conchae, 250μs HF: 25 Hz 0.91(0.43) mA LF: 1 Hz 0.66(0.53) mA 30s on 30s off	Others/ Gastrointestinal problems	ASD	Gastric function **↑**
Wu et al., 2021 (96)	40 patients with primary insomnia Group A: 20 (15 F) Group B: 20 (13 F)	Between repeated taVNS (30min of taVNS twice a day, 5 days per week for 4 weeks)	Group A: 49.40±12.22 years Group B: 46.20±12.76 years	taVNS: cavum concha 7-12mA, 20Hz, 200μs, No stimulation for Group B (controls)	Others/ Sleep problems	GAD MDD	Sleep quality ↑
Zhang et al., 2021 (42)	20 patients with primary insomnia	Within repeated taVNS (30min of taVNS twice a day, 5 days per week for 4 weeks)	Not reported	taVNS: cavum concha 0.8-1.5mA, 4/20 Hz, 200μs,	Others/ Sleep problems	GAD MDD	Score of Pittsburgh Sleep Quality Index **↓** Sleep duration↑
He et al., 2022 (97)	24 patients with chronic insomnia (CI) (12F) 18 healthy controls (HC) (12 F)	Between repeated taVNS for CI patients (30min of taVNS twice a day for 4 weeks),	CI patients: 42.50±15.42 years HC: 43.5 ± 11.23 years	taVNS: bilateral cymba conchae 4/20 Hz, 200μs ± 30% No stimulation for HC	Others/ Sleep problems	GAD MDD	The scores of Pittsburgh Sleep Quality Index and Flinders Fatigue Scale **↓**

F, female; HF, high frequency; LF, low frequency. **↑:** increased/higher/better. **↓:** decreased/lower.

### 3.1 Potential taVNS effects on depression

The common features of pediatric depressive disorders are pervasive sadness, irritability, or anhedonia, along with impairments in a range of cognitive domains such as episodic memory, emotion regulation, sustained attention and capacity for inhibition (98–102). Adolescence is a critical period for the development of depression, and the worldwide prevalence of any depressive disorder in this age group is 2.6% (24). However, around 40% of adolescents with depression do not respond to current psychotherapy or pharmacotherapy interventions and more innovative treatments are needed (103).

The effects of VNS on mood were first observed in patients with epilepsy, and subsequently it was approved for the treatment of refractory depression (104). Several studies have now also used taVNS as a noninvasive alternative of VNS, and found beneficial effects on mood in adult MDD patients (17, 49). Other studies have shown effects on clinical severity. For example, after one month of treatment, scores on the Hamilton Depression Rating Scale were significantly reduced in a taVNS compared to control group in adult MDD patients, and this was associated with increased default mode network functional connectivity under taVNS (13). Kraus and colleagues (38) found that taVNS compared to sham stimulation could decrease BOLD-signals in limbic brain areas and improve subjective well-being ratings (38). Indeed, a range of beneficial taVNS effects have now been reported in a number of clinical trials on MDD patients (14, 15, 17, 49, 105). Recently, evidence from healthy populations also indicates that a prolonged period of effort exertion with concurrent taVNS in comparison to sham stimulation could boost mood recovery, indicating that taVNS may help improve affect after a mood challenge (66).

Previous research has shown that emotion regulation deficits may play an important role in contributing to sustained sad mood in depressive patients (98, 106). In line with this, Koenig and colleagues reported that taVNS decreased attention to sad stimuli in adolescents with MDD when they performed in different emotion recognition tasks (68). Furthermore, in healthy subjects, taVNS reduced the ability to recognize sadness in dynamic bodily expressions (67). Similarly, a recent study indicates that participants receiving active taVNS, compared to sham, were better at using cognitive reappraisal strategy to down-regulate their response to negative emotional pictures (65). Moreover, taVNS could improve impaired cognitive flexibility in depressive patients by enhancing divergent thinking in healthy participants (63). Lack of pleasure (i.e., no interest in reaction to pleasurable stimuli or experiences and lack of anticipation of pleasure) is another main symptom of depression. One recent study demonstrated that acute taVNS facilitated reward-seeking by boosting invigoration, suggesting that taVNS may enhance pursuit of prospective rewards (64). Thus, all the above results suggest that taVNS could be a useful add-on to current therapies for depressive disorders (e.g., emotion regulation, cognitive flexibility, lack of pleasure) in pediatric as well as adult populations.

### 3.2 Potential taVNS effects on anxiety and fear

Anxiety disorders are among the most prevalent psychiatric conditions in children and adolescents worldwide but are often untreated in pediatric populations (107, 108). Excessive fear and anxiety are shared features of anxiety disorders, and uncontrollable and excessive worrying is a typical symptom of generalized anxiety disorder (GAD) in particular (109).

Burger and colleagues suggested that attentional engagement to threat and negative thought intrusions could be reduced by active taVNS in high trait worrying adults, providing preclinical support for future application of taVNS in the treatment of pediatric GAD (69, 70). Fear extinction is also a fundamental step in exposure therapies for anxiety and stress-related disorders (e.g., post-traumatic stress disorder (PTSD)) and low levels of vagal activity have been found in anxiety patients. Thus, VNS could be a non-pharmacological alternative for improving extinction memory (110–112). Studies have now shown that taVNS has beneficial effects on the modulation of fear extinction. For instance, extinction of declarative fear and explicit fear extinction learning could be facilitated by active taVNS compared to sham stimulation (71, 72). Additionally, it has been found that an extinction training together with taVNS resulted in rapid anxiolytic effects as well as an inhibition of fear potentiated startle response (73). Furthermore, associated memory performance and recollection-based memory can be enhanced by taVNS, suggesting its potential role in promoting extinction memory retention beyond its effect on extinction learning (74, 75). Additionally, it has also been found that neurobiological dysfunctions in post-traumatic stress disorder (PTSD), such as increased norepinephrine and sympathetic activity and abnormal inflammatory function, could be modulated by vagal activity (for more detailed discussion see (113)). Thus, taVNS may also be a potential anxiolytic intervention for treatment of pediatric as well as adult anxiety related disorders.

### 3.3 Potential taVNS effects on social dysfunction

Social dysfunction is one of the key characteristics of ASD, and also occurs in ADHD and OCD (114, 115). Impaired emotion recognition is also often observed in these disorders (116). The symptoms of these disorders can often be severe and cause problems in everyday life as well as stress and economic burden for individuals and their families. So far, no effective and reliable treatment has been established for ASD in particular, and there is an urgent need for developing novel effective therapies.

Pre-clinical studies have demonstrated that taVNS can improve emotion recognition in healthy populations. For example, emotion recognition based on the eye region alone (76), whole faces (77) or body movement (67) is enhanced by active taVNS compared to sham stimulation. Further, taVNS can also generally increase emotion recognition in healthy adolescents independent of the type of task (68).Ventura-Bort and colleagues (81) reported that taVNS increased memory performance for emotional but not neutral materials and facilitated early attentional discrimination between emotional and neutral scenes. This may indicate a role of taVNS in increasing the salience of emotional stimuli. In line with this, taVNS has been recently reported to bias visual attention towards salient facial features, which are important for emotional recognition, and increasing endogenous release of the hypothalamic neuropeptide, oxytocin (78). Previously, it had already been found that plasma oxytocin concentrations in rats increased immediately after iVNS (117). A large number of studies have demonstrated as important role for oxytocin in facilitating social cognition and reward (118), taVNS effects on oxytocin may play a key role in helping to increase the salience of social cues (119). Some clinical trials in children with ASD have also shown it can improve social symptoms (120–122). Interoception, which is regarded as a fundamental basis for emotional processing, can also be improved under taVNS, with is evidenced by increased cardiac interoceptive accuracy in a heartbeat discrimination task (79). Furthermore, researchers also found that taVNS modulates attention to direct gaze (salient social cue) irrespective of the expressed emotion in a Rapid Serial Visual Presentation task (80). This finding suggests that taVNS may enhance perception of gaze direction, thereby increasing joint attention, making the observer more sensitive to socially relevant facial cues. In addition, a few studies have reported that massage, which increases vagal activity, can improve social responses and relationships between parents and children with ASD (123, 124). Overall, therefore, the above studies suggest that taVNS has a great potential in improving social cognition and responses (i.e., emotional processing, eye contact) in individuals with neurodevelopment disorders (for more details see **Table 1**).

### 3.4 Potential taVNS effects on impulsivity and inattention

The main features of ADHD are a persistent pattern of inattention and/or hyperactivity-impulsivity that interfere with functioning or development (125). However, impulsive behaviors are also seen in children with ASD and DBD and ones with oppositional defiant disorder (ODD) or conduct disorder (CD). Response inhibition deficits often relate to impulsivity, and together they greatly increase the likelihood that these children will develop antisocial personality disorder or substance use disorders and face incarceration in adulthood (126–128).

Several published meta-analyses of functional MRI studies on ADHD patients have demonstrated abnormal neural activity (129–131) in the executive control and dorsal attentional networks (132–134) which can also be activated by taVNS (50). Other preclinical studies in healthy populations have demonstrated beneficial effects of taVNS on behavioral and executive control, which further suggest its potential therapeutic application in disorders involving problematic impulse control (58). For example, Beste and colleagues (83) investigated the effects of taVNS on different aspects of inhibitory control (i.e., backward inhibition and response inhibition), and reported enhanced response control after active taVNS (83). Subsequently, Fisher and colleagues (2018) demonstrated that taVNS increased adaption to conflict in a response conflict task (the Simon task) (84). Furthermore, response selection during sequential action (85), automatic motor inhibition (135) and self-control in delay discounting (90) have all been reported to be improved by taVNS. It has also been suggested that the effects of taVNS on improving response control in the above studies may be due to its modulatory role in reducing resources required for cognitive control (89). Additionally, emerging evidence has shown that cognitive flexibility, general adaptive control and sustained attention can be enhanced by taVNS, indicating its potential use in alleviating inattention symptoms in pediatric as well as adult ADHD patients (87, 88).

### 3.5 Other clinical symptoms

Children with ASD often suffer from gastrointestinal problems which are associated with vagal activity (136–138). As shown in **Figure 1**, gastrointestinal tract dysfunction could be regulated by stimulating the vagus nerve which plays a key role in the interaction between brain and peripheral organs. Hong and colleagues (93) found that taVNS led to significant reduction in action potential frequency and increased action potential amplitude in the stomach compared to controls, and raised levels of gastrin 3 h after stimulation (93). Subsequently, Teckentrup and colleagues (64) reported that taVNS reduced gastric activity frequency without acutely altering resting energy expenditure (94). A recent study also indicated that gastric motility could be increased by high frequency taVNS (95). These three tentative studies indicate that taVNS may have potential treating of gastrointestinal dysregulations in ASD.

Additionally, a key feature of ASD is restricted verbal and nonverbal communication, and a failure in spoken language development (139). Two recent studies have shown that taVNS could improve novel orthography acquisition and enhance speech category learning in healthy populations. Thus taVNS as an adjunct to language training could be a novel therapeutic strategy for children with ASD (91, 92).

Comorbidity of depression and anxiety in youth is often reported in clinical situations (140, 141), and GAD and MDD have a high rate of comorbidity (142, 143). A possible explanation is that they share some diagnostic symptoms, such as sleeping problems, difficulty concentrating, being easily fatigued, and psychomotor agitation (144). It has been reported that taVNS treatment is effective in improving sleep quality and prolonging sleep duration in primary insomnia patients via the regulation of a broad brain network (i.e., default mode network, salience network and sensorimotor network) (96, 97, 145, 146). He and colleagues (97) have also reported that 4 weeks of taVNS treatment improved chronic insomnia symptoms by decreasing Pittsburgh Sleep Quality Index (PSQI) and Flinders Fatigue Scale (FFS) scores, and increasing the reduced neuroexcitability of the dorsolateral prefrontal cortex. Altered dorsolateral prefrontal cortex excitability was associated with symptom improvements and may therefore predict the efficacy of taVNS treatment effects. Together, this preliminary evidence indicates that taVNS may be expected to play an active role in the treatment sleep problems common in patients with depression and anxiety-related disorders as well as in ASD.

Furthermore, auricular electroacupuncture (EA) on vagally innervated regions, which can mimic taVNS, is reported to be effective in treatment of insomnia and relief of acute and chronic pain as well (147). Recently, Li and colleagues found that taVNS combined with cranial EA can be applied for the treatment of depression with chronic pain (148).

**Figure 2:**
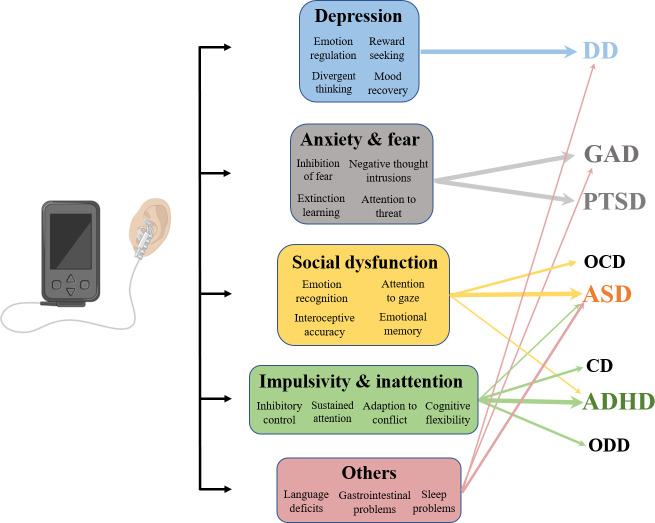
Illustration of the potential effects of taVNS on clinical symptoms and corresponding disorders. Depressive disorder (DD), Generalized anxiety disorder (GAD), Post-traumatic stress disorder (PTSD), Obsessive-compulsive disorder (OCD), Autism spectrum disorder (ASD), Conduct disorder (CD), Attention-deficit/hyperactivity disorder (ADHD), and Oppositional defiant disorder (ODD).

In sum, therefore taVNS may represent a potential therapeutic intervention for a number of different clinical behavioral manifestations targeting pediatric neurodevelopmental and other psychiatric disorders, including ASD, ADHD, OCD, DBD, depression and anxiety-related disorders (see **Figure 2**).

## 4 Optimization of taVNS protocols

Anatomical evidence indicates that the external ear is the only part of our body where the vagus nerve has a peripheral termination (51), and that taVNS produces its functional effects by stimulation of the auricular branch of the vagus nerve (ABVN) (52). Therefore, both the auricular anatomy of vagus nerve and its corresponding physiological properties influence appropriate localization and stimulation parameters for taVNS devices (149), and in turn affect the safety and effectiveness of this technique. Here we detail some of the key factors that need to be considered for optimizing taVNS application protocols in clinical pediatric cases.

### 4.1 Stimulation region

#### 4.1.1 Cymba concha or tragus?

The cymba concha (100% innervated by ABVN) and tragus (45% innervated by ABVN) are the two most frequently chosen auricular regions in taVNS studies (149). However, there is some controversy regarding the optimal positions in the ear for attachment of electrodes for taVNS (150, 151). Notably, it is essential to confirm that it is the vagus nerve rather than other auricular nerves (great auricular nerve, auriculotemporal nerve and lesser occipital nerve) which is activated via taVNS. Evidence from an fMRI study has demonstrated that stimulation of cymba concha induced the strongest activation of the NTS, which is the recipient of most afferent vagal projections located in the brainstem, compared to the ear canal, inner tragus and earlobe (57). Additionally, stimulating the inner tragus relative to the earlobe demonstrated increased activation in brain regions receiving projections from the brainstem (50).The tragus may also have some practical advantages over the cymba concha (150) given that it appears to be easier to apply electrical stimulation by attaching a clip electrode to the tragus rather than by inserting or affixing electrodes to the concha.

Importantly, the current knowledge of auricular vagal nerve anatomy needs to be extended by more anatomical studies on the human ear since to date there is only one dissection study performed on 7 German cadavers (14 ears) (56). Optimal localizations of electrodes need to be informed by more precise future studies.

#### 4.1.2 Left or right ear?

The left ear has been favored most in taVNS studies since it is thought to avoid any risk of incurring possible cardiac arrhythmic effects associated with activation of efferent vagal fibers connected to the right ear (152). However, a study has reported that stimulation of right ear has more beneficial effects on the modulation of heart rate variability (HRV) when compared to left ear (153). A systematic review has also concluded that right-ear stimulation does not increase the risk of aversive effects (154). In addition, bilateral taVNS has been used in a number of studies (64, 155–158) with no obvious adverse events being reported. Currently, studies on the neurophysiological effects underlying different stimulation sites are scarce and more evidence should be provided in future studies, particularly in terms of establishing potential risks in pediatric populations.

### 4.2 Stimulation parameters

The vagal nerve consists of different types of fibers sub-serving specific functions. The myelinated A-fibers which convey somatic afferent information are supposed to be the main target for taVNS (159). Consideration of the signaling properties of Aβ fibers which exclusively send somatic and touch impulses to the central nervous system should be the main focus when deciding optimal stimulation patterns for taVNS. A relatively high frequency of 20-25Hz and short pulse widths are able to recruit thick Aβ fibers (6-12 mm), resulting in activation of the parasympathetic system, while low frequency of 0-0.5Hz and elongated pulse widths are required to stimulate thin fibers, such as myelinated Aδ (1-5 mm) or non-myelinated C fibers (0.4-2 mm), resulting more in activation of the sympathetic system (149).

Currently, there is no consensus on stimulus parameter settings in the taVNS field (61). Variable combinations of frequency, pulse width and intensity have been used given that taVNS devices have been used in a wide range of applications in both clinical and healthy populations (58, 160–162). Although several studies have been carried out to establish optimal stimulation parameters for VNS (163–165), only one has systematically investigated the effects of varying parameters of taVNS (pulse width: 0.1 ms, 0.2 ms, 0.5 ms; frequency: 1 Hz, 10 Hz, 25 Hz) in 20 healthy individuals, and concluded that a combination of 0.5 ms pulse width and 10 Hz frequency induced the greatest effects on heart rate (166). Generally, frequencies of 25 Hz or 20 Hz combined with pulse widths of 0.25 – 1 ms have most commonly been used in previous clinical and preclinical studies (154, 162). In addition, stimulation intensity is often fixed at 0.5mA (37, 72, 82, 83, 167), but in other cases is tailored to individuals' sensitivity/tolerance (50, 57, 153, 168, 169). Furthermore, use of alternating on and off periods of stimulation every 30 s have often been adopted in taVNS procedures to help reduce habituation (63, 77, 85, 88, 170, 171). Overall, therefore, stimulation parameters for taVNS devices still need to be optimized by future studies, particularly for use in pediatric populations.

### 4.3 Stimulation efficacy, side effects and tolerability

Although several studies have tried to investigate the underlying neural mechanisms of taVNS effects, inconsistent findings have been observed due to the variations among stimulation protocols and participants (61). Consequently, no reliable biomarker(s) have been established which could indicate the efficacy of taVNS in general. At present, heart rate variability, some noradrenergic process markers, such as salivary alpha amylase (sAA), P300 amplitude of event-related potentials (ERPs) and pupil dilation are mostly recorded to demonstrate effective vagal activation (for details see review from (172)). However, given the failure of observing increased noradrenergic activity in active taVNS compared to sham stimulation in several studies (86, 167, 169, 173–175), we may need to consider cautiously three possible explanations for the null effects of taVNS. Firstly, suboptimal stimulation parameters. In these studies, pulse width and frequency were kept fixed, although intensity was flexible to adjusted according to individuals pain threshold. However, evidence from animal studies has indicated that it is a combination of intensity and pulse width rather than intensity alone that determines the activation of noradrenergic system (163). Closed-loop taVNS (CL-taVNS) where feedback from rapidly changing bio-signals is used to simultaneously adjust stimulation parameters may be a good choice in future studies to improve treatment efficacy for different disorders (176). Currently, only two CL-taVNS systems exist. The first of these is respiratory-gated auricular vagal afferent nerve stimulation (RAVANS), which works on the principle that inhalation induces transient inhibition of vagal nerve activity, and has shown therapeutic benefits on pain in patients with pelvic pain and migraine (177, 178). A second system is motor-activated auricular vagus nerve stimulation (MAAVNS) (179, 180), which uses electromyography (EMG) to record motor activities as an input signal to guide the administration of taVNS targeting specific motor activity. This is now applied in neonates for oromotor neurorehabilitation (181). In principle, other biomarkers may also be available for developing new CL-taVNS systems in future according to specific clinical purpose. Secondly, unlike invasive VNS that involves the simultaneous activation of afferent and efferent fibers of vagus nerve, taVNS that only stimulates a small branch of afferent vagus nerve fibers may be insufficient to effectively induce measurable central effects on noradrenergic network and the related biomarkers. Thirdly, the earlobe may not be an optimal site to apply sham stimulation given that earlobe stimulation may be associated with the release of other neurotransmitters (e.g., acetylcholine) that also have an impact on the biomarkers of noradrenergic activation (i.e., pupil size, sAA and cortisol). Alternatively, the ear scapha could be a potential site of sham stimulation (182), but central effects of stimulating this site need to be further investigated. Taken together, this also suggests more studies are required to help optimize protocols and stimulation parameters for obtaining reliable results in the future clinical studies.

A systematic review including 1322 participants from 51 studies reported that the most common side effects of taVNS were local skin irritation from electrode placement, headache and nasopharyngitis, although symptoms were usually mild and temporary. Moreover, frequency (Hz) and pulse width (ms) of stimulation were not correlated with the occurrence of side effects (154). In addition, taVNS has been used to treat oral feeding dysfunction in premature newborns (≤33 weeks) (181) and pediatric nephrotic syndrome in young patients (183) without observing adverse events related to stimulation. These suggest that applying taVNS in pediatric populations should represent little risk of significant side effects, although more future trials are included to assess potential short- or longer-term adverse effects.

Tolerance of wearing taVNS electrodes clips in young children, particularly those with ASD, is clearly an issue that needs consideration and it is important that electrode clips are both small and comfortable and that stimulation is not painful. Badran and colleagues have adopted a customized ear-clip the size of which is suitable for newborns to make the taVNS treatment possible (stimulation frequency at 25 Hz, pulse width at 500 μs, and current intensity at 0.1 mA below perceptual threshold) (181). Further, it has also been reported that taVNS could be successfully used in the treatment of pediatric nephrotic syndrome in young children and adolescents (4-17 years, at a frequency of 30 Hz with individual pulse widths of 300 μs, and pulse amplitude intensity was adjusted to the participant's tolerance) (183). However, future studies on children will need to consider use of positive reinforcement to increase cooperation behaviors, adopting CL-taVNS approaches and perhaps in some cases administering taVNS during natural sleep. It is worth noting that many research studies have been performed where young children with disorders are trained to tolerate procedures such as MRI, and to accept wearing EEG or fNIRS electrodes on their head.

## 5 Conclusions

Although research on taVNS has progressively increased in the past two decades, this field is still in its infancy. A number of precautions should be considered for establishing the potential use of taVNS protocols in pediatric populations: (1) More reliable biomarkers of taVNS need to be established, especially the causal link between taVNS and increased vagal activity. Currently, some noradrenergic related activities and parasympathetic functions have been proposed to be the candidates for indicating effective vagus nerve stimulation (i.e., pupil diameter, salivary alpha-amylase and heart rate variability), but inconsistent results have often been reported. Thus, stimulation sites and parameters should be further optimized to enhance treatment efficacy. (2) Long-term and acute effects of taVNS should be carefully investigated, especially for translational purpose, and potential long-term effects need to be investigated in clinical conditions. This information may also help for optimizing individualized treatment. (3) Treatment procedures and outcome measurements can focus on one clinical condition, which may help promote the validation of beneficial effects of the taVNS technique. (4) More preclinical evidence on taVNS effects from pediatric populations is required given that the majority of current studies are from adult populations. (5) The application and side effects of taVNS in young children with neurodevelopment and psychiatric disorders should be investigated in randomized clinical trials. Studies exploring treatment effect of taVNS in children are scarce, and although some have reported no adverse events during the treatment period (181, 183), more future work is urgently needed.

Early intervention is critical to enhancing the quality of life for any child who suffers from symptoms of neurodevelopmental or other psychiatric disorders. For neurodevelopmental disorders in particular there is considerable evidence supporting early therapeutic intervention as having the most effective outcome (184–187) reflecting the fact that developmental changes in the brain are most prevalent at this stage and capacity for brain plasticity changes in response to therapy is highest. In general, taVNS has a tremendous potential as a non-invasive adjunctive treatment targeting specific behavioral manifestations including social dysfunction, impulsivity and inattention, anxiety and fear, and depression in several pediatric neurodevelopment and psychiatric disorders, although standardized stimulation protocols (i.e., stimulation region and stimulation parameters) still need to be established.

## Author contributions

All authors listed have made a substantial, direct, and intellectual contribution to the work, and approved it for publication.

## Funding

This work was supported by the Fundamental Research Funds for the Central Universities, UESTC (grant number ZYGX2020J027 - WZ), China Postdoctoral Science Foundation (grant number 2018M643432 - WZ), Guangdong Basic and Applied Basic Research Foundation (grant number 2021A1515110511 - WZ), Natural Science Foundation of Sichuan Province (grant number 2022NSFSC1375 - WZ), National Natural Science Foundation of China (NSFC) (grant number 31530032 - KK), and Key Scientific and Technological projects of Guangdong Province (grant number 2018B030335001 - KK).

## Conflict of interest

The authors declare that the research was conducted in the absence of any commercial or financial relationships that could be construed as a potential conflict of interest.

## Publisher’s note

All claims expressed in this article are solely those of the authors and do not necessarily represent those of their affiliated organizations, or those of the publisher, the editors and the reviewers. Any product that may be evaluated in this article, or claim that may be made by its manufacturer, is not guaranteed or endorsed by the publisher.
